# An Adaptive Sampling Framework for Life Cycle Degradation Monitoring

**DOI:** 10.3390/s23020965

**Published:** 2023-01-14

**Authors:** Yuhua Yin, Zhiliang Liu, Junhao Zhang, Enrico Zio, Mingjian Zuo

**Affiliations:** 1 School of Mechanical and Electrical Engineering, University of Electronic Science and Technology of China, Chengdu 611731, China; 2 Energy Department, Politecnico di Milano, 20156 Milano, Italy; 3 School of Electrical and Electronic Engineering, Nanyang Technological University, Singapore 639798, Singapore; 4 Centre de Recherche sur le Risque et les Crises, MINES Paris—PSL University, 75006 Paris, France; 5 Qingdao International Academician Park Research Institute, Qingdao 266041, China

**Keywords:** data redundancy, data loss, condition monitoring, adaptive sampling strategy, mechanical degradation monitoring

## Abstract

Data redundancy and data loss are relevant issues in condition monitoring. Sampling strategies for segment intervals can address these at the source, but do not receive the attention they deserve. Currently, the sampling methods in relevant research lack sufficient adaptability to the condition. In this paper, an adaptive sampling framework of segment intervals is proposed, based on the summary and improvement of existing problems. The framework is implemented to monitor mechanical degradation, and experiments are implemented on simulation data and real datasets. Subsequently, the distributions of the samples collected by different sampling strategies are visually presented through a color map, and five metrics are designed to assess the sampling results. The intuitive and numerical results show the superiority of the proposed method in comparison to existing methods, and the results are closely related to data status and degradation indicators. The smaller the data fluctuation and the more stable the degradation trend, the better the result. Furthermore, the results of the objective physical indicators are obviously better than those of the feature indicators. By addressing existing problems, the proposed framework opens up a new idea of predictive sampling, which significantly improves the degradation monitoring.

## 1. Introduction

**Figure 1:**
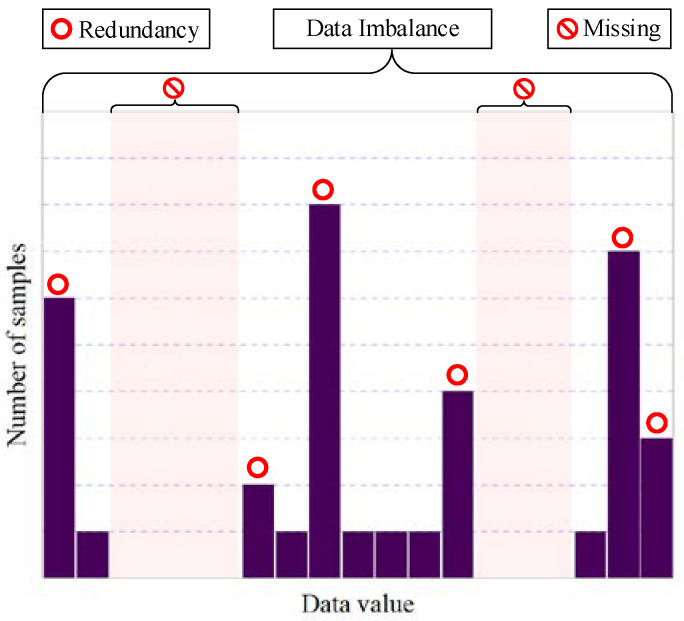
Data problems in condition monitoring.

Condition monitoring (CM) began with machinery [1] and has gradually expanded to a wide range of applications, including healthcare [2], climate environment [3], etc. Real CM data are generally collected by adopting a time-based sampling strategy, which suffers from prominent data problems, including the challenges of data redundancy and data loss. Data redundancy increases the pressure of data storage, the consumption of data transmission and the efforts of data processing; data loss results in the loss of condition information and affects the performance of the built model. In addition, as illustrated in Figure 1, these can also cause a data imbalance problem [4,5]. Currently, most studies are devoted to solving these data challenges from a methodological perspective. From a statistical viewpoint, it is almost always better to have more observations. Although some resampling methods have taken into account the perspective of data [6,7], they just reuse or reconstruct the original data, but do not fundamentally solve the original data problems. In fact, these problems are relevant issues, and the sampling strategy can address them from the source of data.

### 1.1. The Sampling Strategy and Its Control Variables

**Figure 2:**
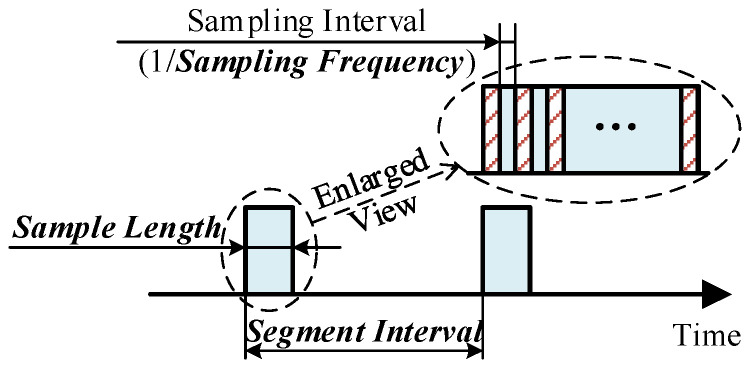
Illustration of three shared control variables.

In CM, data acquisition is determined by the sampling strategy, and the selection of control variables is the key to realize sampling strategy optimization. In this paper, control variables are classified into two categories: exclusive variables and shared variables. The former refers to variables unique to a specific scenario, including the variable types of sampling objection [8], sensor types [9], sensor number [10], installation method [11], etc. The latter denotes variables included in any scenario, as illustrated in Figure 2, it consists of segment interval, sample length, and sampling frequency.

Shared variables are a shortcut to solve existing problems. Among them, the sampling frequency has received the most attention. This, however, is only effective for data redundancy, and does nothing to help with the data loss. The segment interval can cope with them both, but much less relevant research has been conducted.

### 1.2. A Sampling Strategy Based on Tuning the Segment Interval

**Figure 3:**
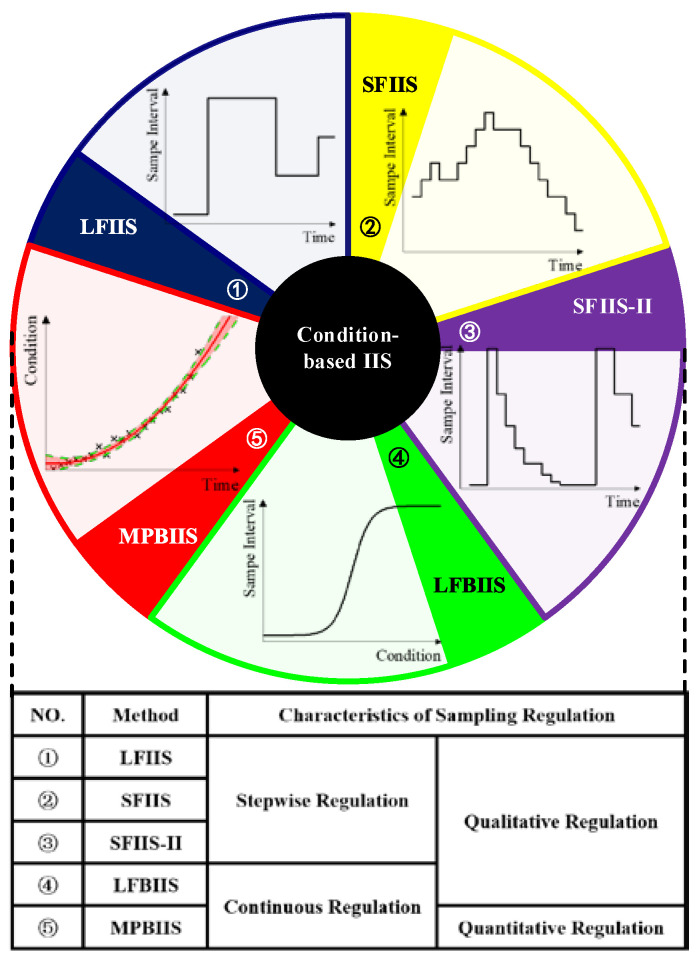
A sketch map and the characteristics of Condition-based IIS.

The traditional time-based strategy samples data with a fixed segment interval. Due to a lack of adaptability to data, condition-based approaches have been explored by regulating the segment interval dynamically as actual conditions change. This can also be called irregular interval sampling (IIS). Local-fixed IIS (LFIIS) performs different time-based strategies in different conditions, and it can be realized via condition classification, condition identification and segment determination [12,13]. As LFIIS cannot adapt to condition changing, step-fixed IIS (SFIIS) has been developed for increasing or decreasing intervals to efficiently cope with condition fluctuations [14,15,16,17]. For fast degradation, scale-fixed IIS (SFIIS-II) works better than SFIIS, as it can achieve multiplicative adjustment [14,18] and even roughly minimize the segment interval to avoid information loss [18,19]. As illustrated in Figure 3, the three strategies above can be visually interpreted as stepwise methods, and they account for the largest proportion of existing studies. Nevertheless, they are triggered methods, and the gaps between their steps inescapably become an obstacle to possible ideal sampling. A continuous regulation method can resolve these gaps by constructing successive functions to associate the condition to the segment interval: it is, however, difficult to find a precise function for describing their relationship. Settling for second-best, logical function-based IIS (LFBIIS) utilizes a logically correct function to represent this relationship via an explicit or implicit function [19,20,21,22].

**Figure 4:**
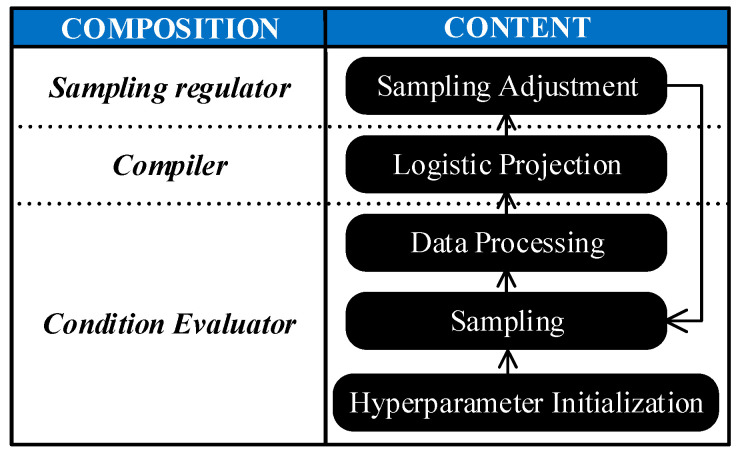
General scheme of condition-based IIS strategy.

The process of condition-based IIS has been summarized in Figure 4. A condition evaluator is responsible for obtaining condition information, a compiler is responsible for converting condition information to a theoretical segment interval, and a sampling regulator is responsible for generating the final sampling strategy by synthesizing all information. No matter whether a stepwise regulation method or LFBIIS is employed, however, there is only a qualitative correlation between the monitored condition and the segment interval, as no rigorous mathematical derivation has been shown. To obtain the ideal sample, the most promising method is to discover the mathematical relationship between them, i.e., to employ mathematical prediction-based IIS (MPBIIS). A comparison table of different types of sampling strategies on segment interval is summarized in Table 1. The types of sampling strategy in the table are represented by a circled number, where the time-based strategy is represented by a circled 0, and the other types refer to Figure 3.

At present, the following four problems hinder the realization of MPBIIS, and are identified as P1~P4.

P1: Ambiguous sampling target: the ambiguity inevitably leads to a theoretical deviation from the potential ideal sampling. Although attempts have been made by mathematical prediction [23], remedies can only be carried out from the perspective of error control, due to the absence of a clear target.

P2: Irregular time series prediction (ITSP): as real data tend to be nonlinear and unrepeatable, predictions based on such data belong to the ITSP problem, which remains unsolved in a wide range of fields.

P3: Time-lagging nature: existing methods adjust the sampling of next moment according to the condition of previous moment, which does not take the condition’s time-varying nature into account.

P4: The prediction of segment interval: time series forecasting is dedicated to predicting condition at a given time, which is exactly the opposite of our needs for sampling.

Considering the huge difference in data characteristics, it is impossible to find a universal solution. One practical solution is to explore domain-specific methods by focusing on a specific scenario. Following this idea, an adaptive sampling strategy framework is proposed aiming at the issue of degradation monitoring. The contributions of the work are given below.

(1) We firstly define and summarize the control variables of sampling strategy methods, and review the research related to sampling strategies on segment interval.
(2) We propose a new framework for degradation monitoring. This framework is further implemented in mechanical degradation monitoring and can apparently improve or even eliminate existing problems.
(3) We advance a new scheme to evaluate the data problems in CM from three perspectives with five metrics.

## 2. Methodology

Degradation is widespread in nature. Its evolution in time makes the individual gradually tend toward a tipping point [24], while this transition to a state of failure can have catastrophic consequences. To avoid the huge risks caused by degradation accumulation, much attention has been paid to sensor development and data usage to detect, diagnose and predict degradation. Such research focuses on information acquisition and mining, whereas the issue of the sampling strategy is undervalued, especially concerning the segment interval.

### 2.1. The Proposed Framework for an Adaptive Sampling Strategy

**Figure 5:**
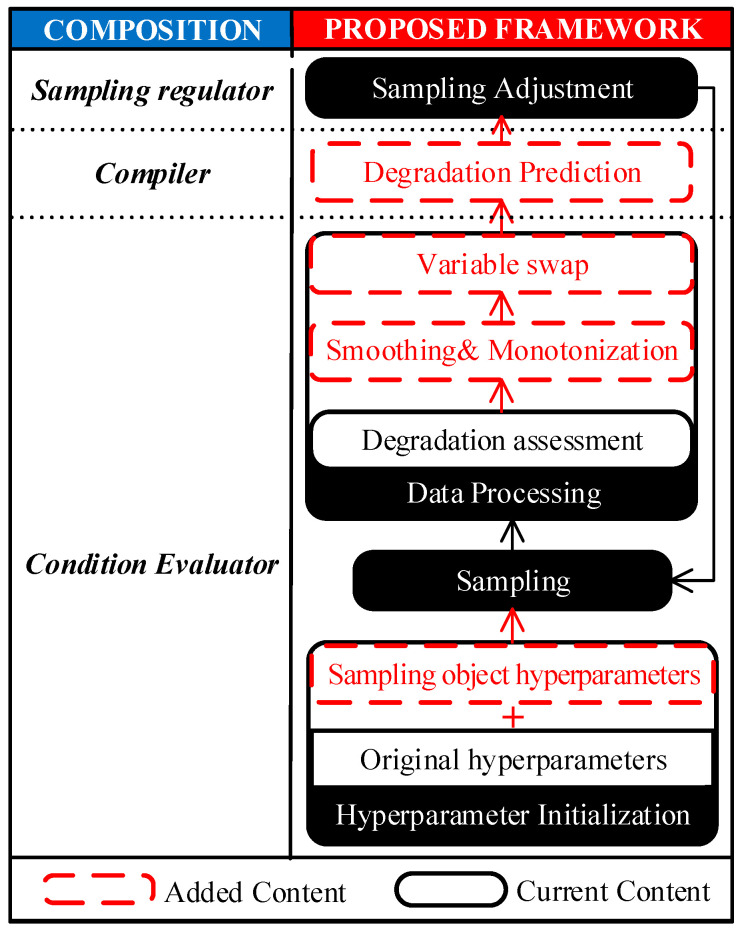
A diagrammatic sketch of the proposed framework.

In response to the four problems of segment interval, an adaptive sampling framework is proposed by adding targeted content to deal with the issue of degradation. Increments are marked by red dashed lines in Figure 5.

#### 2.1.1. Hyperparameter Initialization

Except for traditional hyperparameters, those related to the distribution of sampling objects are introduced to clarify the sampling needs in P1, and they involve the target segment interval and target sample quantity. The target segment interval refers to the degradation indicator intervals that are wanted, and the target sample quantity denotes the desired sample number of each target segment interval.

The most widely used distribution is the uniform distribution, which has a target sample quantity of 1 in each interval and equal length target segment intervals. In this case, only the degradation interval needs to be specified. In addition, considering that the influence of the same degradation amount may be different in different conditions, just as people have different sensitivity to pain at different ages, these distributions must be determined on a case-by-case basis.

#### 2.1.2. Time Series Collection

The collection of the initial time series relies on an initial sampling strategy, whereas the subsequent sampling is based on the results of condition predictions in the sampling regulator.

#### 2.1.3. Transforming from a Time Series to a Degradation Series

The main difference between the proposed framework and traditional strategies lies in the increment operation of the variable swap, i.e., converting the time series to a degradation series. Time series prediction enables numerical inferences at specific future times. It, however, cannot estimate the time at a specific numerical value of degradation. To eliminate the theoretical error of time estimation in P4, a variable swap is designed to exchange the independent and dependent variables of the time series.

Before this conversion, the timesseries must be monotonic, a condition that is satisfied in most cases, since degradation itself is irreversible. For the fluctuations caused by noise and measurement error, smoothing and monotonization can be used to calibrate them to approximate actual degradation.

#### 2.1.4. Degradation Prediction

As most degradation laws are nonlinear, regardless of whether the collected samples form an irregular time series or not, the obtained degradation series is commonly an irregular series after the variable swap is conducted, and its prediction is an ITSP issue of P2.

One possible approach is to transform the irregular series to a regular series and utilize times series forecasting methods for prediction. This would be best for predicting irregular series directly without transformation error. However, existing solutions of this sort are only applied in a few areas, such as astronomy [25]. The feasibility of existing ITSP methods can be explored for specific datasets. In addition, machine learning methods are very promising options. Meanwhile, the realization of prediction settles the time lag problem of P3.

#### 2.1.5. Segment Interval Calculation

After obtaining the forecasted segment interval, the actual segment interval still requires the consideration of certain time boundaries to avoid possible surprises, as the laws on which our predictions are based may change or even mutate. These boundaries should be valued in Section 2.1.1. Subsequently, based on the actual segment interval, a new sample should be obtained in a loop until the failure threshold is reached.

### 2.2. A Proposed Method for Mechanical Systems

Mechanical equipment is pervasive in industry and a great deal of effort has been dedicated to its reliability and safe operation. At present, condition-based maintenance (CBM) is the state-of-the-art solution for counteracting the influence of mechanical degradation on reliability and safety. The realization of CBM includes the following three steps [26,27]: data acquisition, data processing method and maintenance decision-making. The latter two have received the most attention. Taking the popular statistical learning techniques as the example, many methods has been applied in degradation monitoring, including supervised learning [28], unsupervised learning [29], transfer learning [30], statistical model [31], integrated learning [32], etc. For existing data problems, a large number of studies have also been carried out to reduce their impacts in the tasks of classification [33,34] and regression [5,35]. However, they all deal with this problem from the perspective of methodology, and the solutions from a data perspective have been underestimated. Considering its significance in practice, mechanical degradation monitoring was chosen as the object of the framework’s application.

**Figure 6:**
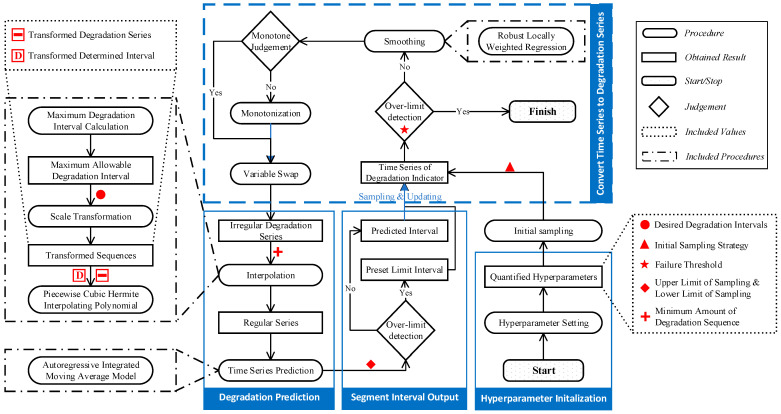
Flow chart of IIS method for mechanical degradation monitoring.

Combined with the specific characteristic of mechanical degradation, a concrete method is advanced based on the proposed framework. The method can be applied to available monitoring data in numerical data formats, including pressure, temperature, acoustic emission, wear amount, voltage, current, etc. The applicability of the method depends on whether the data type can effectively reflect the condition degradation, which needs to be determined according to the specific scenarios. Since it is implemented based on the condition prediction, the most beneficial conditions are closely related to the predictability of the degradation, as follows: (1) The degradation indicator can effectively represent the degradation; (2) the degradation process is stable; (3) the degradation law is consistent and no mutation occurs. The method flow is illustrated in Figure 6.

Step 1. Hyperparameter initialization. The first step is the determination of the target sampling distribution. Generally, the existing condition-based methods tend to accelerate or reduce sampling, for poor or good health conditions. Although not explicitly mentioned, the logic behind this is to make the difference of adjacent samples as small as possible. In other words, the implied target sampling is the uniform distribution. Occasionally, this target sampling may be a non-uniform distribution, which needs to be determined specifically.

In addition, the initial sampling strategy and sampling boundary should be specified. The former generally adopts a time-based strategy for the data accumulation process before the loop. For the latter, the upper limit of sampling is to prevent a sudden change of the condition law as well as possible large errors in prediction, and the lower limit of sampling is to avoid the sampling moment of the prediction being earlier than the moment when the prediction is completed.

Step 2. Convert the time series to a degradation series. After obtaining the samples, we need to check whether they have reached the failure threshold or not. If yes, the sampling should be finished and an output should be obtained from the samples. Otherwise, smoothing the time series via robust locally weighted regression can balance the trend and outliers well, and is especially useful for the robust handling of outliers. If the time series does not satisfy monotonicity at this time, additional monotonic processing is required. Finally, the independent variable and dependent variable of the time sequence must be exchanged to obtain the degradation series.

Step 3. Degradation series prediction. After the variable swap, the obtained sequences are basically irregular series. In this method, the regular time series forecasting method is selected to realize irregular time series forecasting, on the basis of interpolating irregular time series to obtain a regular series. Before this, the minimum account of the degradation series *V*_1_ must be set to meet the data volume requirement for prediction. Accordingly, the maximum allowable degradation interval can be calculated:

(1) $$MADI=\left(h_{last}-h_{1}\right)/V_{1}$$

where *MADI* is the abbreviation for the maximum degradation interval, and *h*_1_ and *h_last_* are the first and last items, respectively.

On this basis, an actual segment interval can be determined and further utilized for scale transformation with Equation (2), that is, the segment interval selection and degradation series transformation.

(2) $$SI_{s}=min(SI_{d},MADI),\;S_{t}=S/SI_{s}$$

Hereere, *SI_s_* and *SI_d_* are the selected and target segment intervals, respectively. *S* and *S_t_* respectively indicate the sequence before and after transformation. and *min*( ) is the minimum function.

Let the transformed degradation sequence be expressed as {*th_i_*: *i* = 1, 2, …, *n*} and construct a series of interpolation points {*H_j_*}. Since the degradation sequence was normalized by *SI_a_* via scale transformation, the actual segment interval has been converted to the unit length in {*th_i_*}. Consequently, {*H_j_*} is an arithmetic sequence, whose last term is *th_last_*. The difference is 1 and *j* = *floor*(*th_last_* − *th*_1_), where *floor*( ) only outputs the integer part of the value in parentheses. Then, the piecewise cubic Hermite interpolating polynomial is selected to obtain the regular series. This can preserve the data’s shape and corresponding monotonicity, which is exactly what we want. For a subinterval [*h_k_*, *h_k_*_+1_], let

(3) $$l_{k}=h_{k+1}-h_{k},\;d_{k}=\left(t_{k+1}-t_{k}\right)/l_{k},\;s_{k}=F^{\prime }(h_{k})$$

where *s_k_* is the slope of point *h_k_* equal to *d_k_* or *d_k_*_+1_ for the piecewise linear interpolation. The fitted cubic polynomial *F*(*h*) can be represented as follows, for *h_k_* ≤*h*≤ *h_k_*_+1_:

(4) $$F(h)=\frac{3l_{k}\Delta^{2}-2\Delta^{3}}{l_{k}{}^{3}}t_{k+1}+\frac{l_{k}{}^{3}-3l_{k}\Delta^{2}+2\Delta^{3}}{l_{k}{}^{3}}t_{k}+\frac{\Delta^{2}(\Delta -l_{k})}{l_{k}{}^{2}}s_{k+1}+\frac{\Delta {\left(\Delta -l_{k}\right)}^{2}}{l_{k}{}^{2}}s_{k}$$

(5) $$\Delta =h-h_{k}$$

Bringing {*H_j_*} into *F*(*h*) on the corresponding subintervals to obtain the new time sequence {*T_j_*}, then, the time series {(*t_j_*, *h_j_*)} is transformed into a degradation series {(*H_j_*, *T_j_*)}.

Afterwards, the autoregressive integrated moving average is utilized for sampling time prediction, which has been widely adopted and proven effective for mechanical degradation processes. This model can be represented by ARIMA(*p, d, q*), where *p* is the lag order that denotes the number of lag observations in the model; *d* is the differencing degree that refers to the number of times the raw observations are differentiated; *q* is the moving average order, which means the size of the moving average window. The model is expressed as follows:

(6) $$\varphi (B){\left(1-B\right)}^{d}T_{t}=\theta (B)\varepsilon_{t}$$

where *ɛ_t_* is the random error at time *t*; *φ*( ) is a function of a *p*-order autoregressive coefficient polynomial, and *θ*( ) is a function of a *q*-order self-moving average coefficient polynomial, and they are expressed by Equation (7):

(7) $$\varphi (B)=1-\sum_{i=1}^{p}\varphi_{i}B^{i},\;\theta (B)=1+\sum_{i=1}^{q}\theta_{i}B^{i}$$

Hereere, *B* is the backshift operator defined as

(8) $$BT_{t}=T_{t-1}$$

where *T_t_* and *T_t_*_-1_ represent the *t*^th^ and (*t*-1)^th^ element in {*T_i_*}.

The Box–Jenkins methodology is utilized to set up an ARIMA model that only needs a one-step prediction to forecast the sampling time of the target segment interval.

Step 4. Segment interval output. The upper and lower sampling limits are denoted as *I*_max_ and *I*_min_, and the actual segment interval is decided as follows:

(9) $$SI_{a}=\left\{\begin{array}{l} I_{\max},I_{\max}\leq SI_{p}\\ SI_{p},I_{\min}\leq SI_{p}\leq I_{\max}\\ I_{\min},SI_{p}\leq I_{\min} \end{array}\right.$$

where *SI_a_* denotes the final segment interval and *SI_p_* is the predicted interval. In this way, we can sample with *SI_a_* and update the sample set of the time series. Afterwards, we return to Step 2 and loop until the termination condition is reached.

## 3. Experimental Validation and Discussion

Simulation and real experimental data are used to test the performance of the proposed sampling method. All of the experiments are performed on a laptop with the Windows 11 operating system, an Intel Core i5-10210U CPU and 8 GB memory.

### 3.1. Comparison Experiment Setup

Three comparison methods and five performance metrics are introduced to verify the effect of the proposed method.

#### 3.1.1. Comparison Methods

As a representative stepwise method, SFIIS-II is selected as the first comparison method, of which the degradation rate is chosen to adjust the sampling and defined as follows [14,18]:

(10) $$Rate=\frac{\Delta HI}{\Delta t}$$

where ᐃ*HI* and ᐃ*t* represent the change of degradation indicator and time.

A big scale∈(1, +∞) is used to multiplicatively adjust the segment interval for slow degradation, and a small scale∈(0, 1) is used for fast degradation. Otherwise, the interval stays unchanged.

As the only strategy of continuous regulation, LFBIIS is selected as another comparison method. Its logistic function is a variant of the sigmoid function [20]. The segment interval is represented by its abbreviation *SI*, and the reciprocal of *SI* can be regarded as the horizontally transposed sigmoid function of the *Rate*. Let *y* = 1/*SI*, the logistic function expressed as follows.

(11) $$y(Rate)=y_{\min}+\frac{\left(y_{\max}-y_{\min}\right)}{1+e^{-coef*(Rate-l_{hm})}}$$

where *y_min_* = 1/*I_max_*, *y_max_* = 1/*I_min_* and *l_hm_* represents the horizontal moving length of the sigmoid function. The term *coef* is a coefficient to adjust the speed of change rate.

#### 3.1.2. Performance Metrics

Five metrics are designed to assess the sampled data; see Table 2. In addition, since a short execution time is important for sampling adjustment, fixed model parameters are chosen to reduce model prediction time, and the execution time for a single prediction is given subsequently. For all the three types of data, the prediction model of arima(1, 2, 0) works well and is adopted for prediction. In the model, the lag order *p* equals to 1, which indicates that only the previous value in the process and the noise contribute to the prediction result. In addition, a second order difference is applied to eliminate a quadratic trend of data. The differencing degree *d* is greater than 0, which reflects the non-stationary nature present in the experimental data.

Assuming the initial sample set is {*x_i_*, *i* = 1, 2, …, *p*}, the set of target segment intervals {*S_m_*} can be constructed by using *SI_d_* as the length of the sub-intervals. Let *I_j_* express the *j*^th^ sub-interval, {*S_m_*} = {*I_1_* U *I_2_* U… U *I_m_* | *I_j_* = [*x_p_* + (*j −* 1/2) × *SI_d_*, *x_p_* + (*j*+1/2) × *SI_d_*)}. Then, the obtained samples {*X_i_*, *i* = 1, 2, …, *n*} can be judged by the data distribution in {*S_m_*}. All the metrics are relative values for ease of comparison.

(12) $$\rho_{t}=\frac{n}{IN_{t}},\;\rho_{r}=\frac{n}{IN_{t}-IN_{l}},\;R_{r}=\frac{IN_{r}}{IN_{t}},\;R_{l}=\frac{IN_{l}}{IN_{t}}$$

(13) $$D_{a}=\frac{1}{n}\sum_{i=1}^{n-1}\left|X_{i}{}_{+1}-X_{i}-SI_{d}\right|$$

where *IN_l_*, *IN_r_* and *IN_t_* refer to the numbers of loss sub-intervals, redundancy sub-intervals and total sub-intervals, respectively. 

### 3.2. Simulation Data

**Figure 7:**
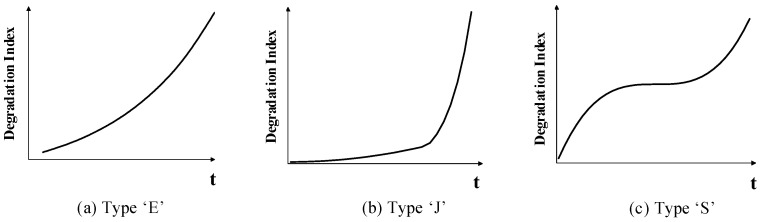
Three typical mechanical degradation curves.

We conclude three typical forms of mechanical degradation curves, as shown in Figure 7. The first, type “E” is the most used exponential degradation model with a single degradation law. The types “J” and “S” are used to simulate two-stage and three-stage degradation laws, respectively. Since the simulation data are a continuous numerical simulation, the available data points are theoretically infinite, and the actual data obtained are calculated according to the predicted sampling time.

**Figure 8:**
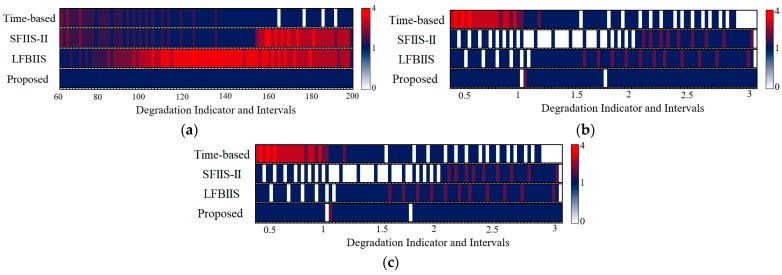
The distribution of sampled data from simulation data. (**a**) Type “E”; (**b**) Type “J”; (**c**) Type “S”.

The distributions of the sampling results are shown in Figure 8, and the color bar on the right indicates the collected points number of each interval in {*S_m_*}. It can be seen that the time-based strategy tends to lose some information in rapid degradation and gains redundancy in slow degradation. The scale-based and LFBIIS strategies improve upon both of these problems to some extent, but they also sometimes have side-effects. For instance, information loss appears when they try to reduce the redundancy in (b), and information redundancy is caused when reducing the information loss in (a) and (c). The reason behind this lies in the fact that they are essentially qualitative methods. Due to the constant scale coefficients of scale-based strategy, different collection volumes result in different conditions. Additionally, although the LFBIIS strategy seems more flexible to different conditions, it is impractical to find a universally applicable function to fit a changeable degradation law. Compared to their unstable performance, the proposed strategy achieves near perfect performance as a whole. Although there is still information loss, and although redundancy appears when conditions change, perfect sampling can be restored quickly.

All the result analyses above are shown in the quantitative metrics of Table 3, and the average execution time for a single prediction is 0.0392 s. The first row are ideal values that denote the best values of certain degradation laws. Consistent with the previous analysis, the performance of the proposed strategy surpasses the others in almost all items. The only parameter that does not seem to be optimal is the amount of data under “S” type degradation. The best performance of *ρ_t_* is, however, a source of massive information loss, which makes this advantage meaningless. Therefore, the proposed strategy achieves a state-of-the-art performance on all datasets.

### 3.3. Real Experimental Data

As the quality, volume and type of real experimental data have considerable influence on the implementation of a sampling strategy, available public datasets on mechanical degradation are investigated, and the results are shown in Table 4. The time-based sampling strategy is the mainstream sampling strategy, and bearings are the most widely researched subject.

For real experimental data, degradation indicator acquisition is both critical and challenging. Available options consist of physical indicators and feature indicators, and both of them can be utilized in cutter degradation measurement. By contrast, bearings and engines are hard to monitor by direct physical indicators, but feature indicators can be extracted from indirect measurements, such as vibration and temperature. To test the performance of these two types of indicators, cutter data are the choice for the physical indicator, and bearings are chosen as the feature indicator.

The main challenge in using real experiment data is data discontinuity, which hinders the accurate acquisition of data at desired time. The more data there are, the less influence this may have. As such, the datasets of PHM2010 and FEMTO-ST are selected for validation, and the closest point available is used as the actual sampling time.

#### 3.3.1. Physical Indicator Case

The PHM2010 dataset was collected on a high-speed CNC machine with a fixed segment interval of each tool walk. After each tool walk, the wear amount of three cutting edges were measured with a microscope. Six batches of data are captured from six cutters, half of which are marked with labels for wear amount and can, thus, be used for the experiment: this includes Cutter#1, #4 and #6. Each batch of data collected 315 samples, and each sample records the wear amount on the three edges of the cutter. Suppose that the segment interval of adjacent samples is 1, then the interval of a time-based strategy is set to 2 to give irregular strategies a chance to obtain more compact sampling. The average wear amount of three edges is set as the physical indicator value of tool degradation.

**Figure 9:**
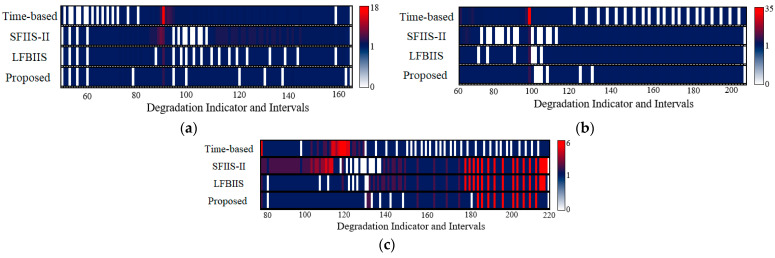
The distribution of sampled data from the PHM2010 dataset. (**a**) Cutter #1; (**b**) Cutter #4; (**c**) Cutter #6.

The sampling results are shown in Figure 9. Compared with the simulation data, more data loss appears in all strategies, which is caused by the limited data volume. Still, the proposed strategy obtains the least amount of loss. When we focus on sample value, the deviation between segment interval and the ideal value is also much greater than in the simulation data, which not only relates to the restriction of data volume but also involves the influence of data fluctuations to condition forecasting. Although the ideal sampling is unachievable based on the available data, the proposed strategy still tries to approach the ideal value by adjusting the segment interval, whereas other strategies do not. Referring to the metrics in Table 5, the performance of the proposed strategy far exceeds the others, and the average execution time for a single prediction is 0.0363 s.

#### 3.3.2. Feature Indicator Case

The FEMTO-ST dataset used in this section was gathered on the PRONOSTIA platform. During the rotation of the bearings, a radial force is applied by a pneumatic jack and adjusted by a digital electro-pneumatic regulator. Vibration data are collected by miniature accelerometers with a frequency of 25.6 kHz. Three conditions are designed to simulate different working conditions with multiple experiments under each condition. To ensure as much degradation data as possible, Bearings 1_1, 1_3, and 1_4 are chosen as candidates. Their sample point numbers are 2803, 2375, and 1428, respectively; each sample records the vibration data of three directions, and each direction includes 2560 points. Root mean square values are extracted as the feature indicator, and only the degradation data are selected for experiments.

**Figure 10:**
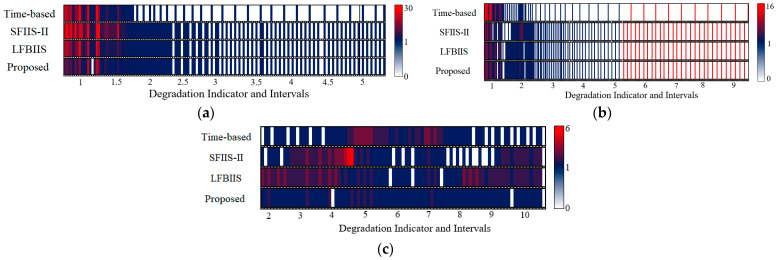
The distribution of sampled data from real bearing data. (**a**) Bearing 1_1; (**b**) Bearing 1_3; (**c**) Bearing 1_4.

The graphical results of sample distributions are shown in Figure 10. Compared with the previous experiments, the amount of information loss and distribution imbalance significantly increases in Bearings 1_1 and 1_3. Although the metrics of the proposed strategy in Table 5 still dominate, the advantage is negligible or even inferior to that of other strategies. By contrast, the application on Bearing 1_4 shows extremely good performance, in which all the metrics have a clear advantage over the other strategies, which stems from a discrepancy in the data volatility. There are many fluctuations in the first two sets of data, whereas the last set of data is relatively smooth. The fluctuations create difficulties for condition forecasting, which seriously impacts the effect of the proposed strategy. In addition, the average execution time for a single prediction is 0.0376 s.

### 3.4. Summary

Synthesizing the performances on experimental data, there is no doubt that the proposed method obtained the best results. Its performance on different datasets varied, showing that:

(1) Data status and degradation indicator are critical to the performance of the sampling strategy, and the influence factors include the data fluctuation, data volume, the selection of the degradation indicator, the stability of the degradation trend, etc.;
(2) Abrupt changes in degradation greatly influence the sampling result, no matter which strategy is chosen. This serves as a reminder to maintain a safety margin in sampling strategy formulation to avoid possible information loss;
(3) Sampling strategies based on feature indicators are still of great significance, combining their dominant position in mechanical CM. Although their performance in the experiments is the worst, feature indicators still have huge potential. They are already able to properly describe the degradation process in many scenarios, as seen, for example, with the intelligent algorithms that have emerged in recent years and, especially the superior performance of deep learning methods in feature extraction;
(4) As well as improving the condition indicator, the methods of irregular series prediction can also be introduced to sampling optimization. The series conversion of the proposed method inevitably introduces errors. Thus, direct prediction with irregular series may be of promise in promoting sample quality;
(5) Real experimental data come from public datasets, which restricts the full realization of sampling strategies. Research on sampling strategies of the segment interval is still in its infancy. If dedicated public datasets can be built, they will greatly promote the development of related research;
(6) Reasonable strategy selection and parameter setting are necessary to avoid sampling results being affected, or even worse than those of time-based sampling.
(7) The average execution time for a single prediction is less than 0.04 s, which shows that the proposed method has good work performance in a real-time scenario.

Considering the fact that the degradation law and data status are objective factors, the selection of the sampling strategy type is crucial to the sampling results. For sudden faults, SFIIS is the most suitable type due to its fastest sampling adjustment, including the faults of component shedding, fatigue fracture, unbalance and misalignment during operation, etc. For degradation faults, the most suitable type is closely related to the characteristics of degradation indicators, and can be selected according to the summary of different types of strategies shown in Table 1, such as the faults of wear, peeling, corrosion, cracks, etc. With the development of predictive technologies, the proposed framework will become more and more perfect and promising. Four benefits and two challenges of the proposed method are summarized in Table 6.

## 4. Conclusions

CM suffers from the problems of data redundancy and data loss, and sampling optimization is expected to solve these at the source. As a key part of the sampling strategy, the segment interval choice has not received due attention and lacks extensive research. This paper proposes a new framework to improve degradation monitoring with respect to existing problems of segment interval. The proposed framework is implemented on the CM of mechanical degradation by specifying methods, including adding sampling object hyperparameters in the module of hyperparameter initialization for the problem of sampling targets ambiguity, applying the interpolation method of the piecewise cubic Hermite interpolating polynomial to solve the issue of ITSP, adopting the prediction method of the autoregressive integrated moving average model to answer the lagging nature of available solutions, and designing a variable swap to resolve the error problem caused by inaccurate estimation of the sampling time. Subsequent experiments showed the comprehensive superiority of the proposed method over comparable methods. Through the improvements to existing problems, the proposed method greatly alleviates or even eliminates the influences of data redundancy and data loss. The selection of the degradation indicator is a key factor affecting the effect of sampling strategy implementation. As the research on segment interval sampling strategy is still in its infancy, it still has huge potential for further improvement.

## Figures and Tables

**Table 1:** Comparison table of different types of sampling strategies on segment interval.

No.	Literatures	Benefits	Challenges
⓪	-	Easy to implement	Data redundancy and data loss
①	[12,13]	Improves the two data problems	Lacks of adaptability to changing conditions
②	[14,15,16,17]	Adaptable to changing conditions	Inability to cope with large condition changing
③	[18,19]	Responds quickly to large condition changing	Sampling gaps caused by stepwise adjustment
④	[19,20,21,22]	Continuous adjustment without sampling gaps	Qualitative adjustment with principle error
⑤	-	Quantitative adjustment without principle error	Uncertainty risks caused by forecasting

**Table 2:** Performance metrics and their representation.

Problems	Performance Metrics	Symbol
Data redundancy	Total density of sample	*ρ_t_*
Data redundancy	Redundancy density of sample	*ρ_r_*
Data redundancy	Rate of information redundancy	*R_r_*
Data loss	Rate of information loss	*R_l_*
Sampling deviation	Average deviation	*D_a_*

**Table 3:** The results of performance metrics for simulation data.

	*ρ_t_*	*R_m_*	*ρ_r_*	*R_l_*	*D_a_*	
Ideal Value	1	0	1	0	0	
Time-based	1.29	0.04	1.35	0.30	0.72	Type “E”
SFIIS-II	2.37	0	2.37	0.63	0.87	
LFBIIS	3.86	0	3.88	0.91	1.12	
Proposed	1	0	1	0	9 × 10^−4^	
Time-based	1.26	0.22	1.61	0.25	0.015	Type “J”
SFIIS-II	0.74	0.36	1.16	0.10	0.018	
LFBIIS	1.05	0.08	1.14	0.13	0.006	
Proposed	0.99	0.02	1.01	0.01	9 × 10^−4^	
Time-based	1.06	0.39	1.73	0.13	0.0194	Type “S”
SFIIS-II	2.11	0.22	2.71	0.48	0.0186	
LFBIIS	1.91	0.07	2.06	0.57	0.0141	
Proposed	1.11	0.06	1.18	0.06	0.0048	

**Table 4:** Some available public datasets.

Name	Data Type	Research Object
FEMTO-ST	Regular Series	Bearing
PU	Regular Series	
XJTU-SY	Regular Series	
IMS	Regular Series	
SJTU	Irregular Series	
PCoE-Milling	Irregular Series	Cutter
PHM2010	Regular Series	
PCoE-PHM08	Regular Series	Engine

**Table 5:** Results of performance metrics for the PHM2010 dataset.

	*ρ_t_*	*R_m_*	*ρ_r_*	*R_l_*	*D_a_*	
Ideal Value	1	0	1	0	0	
Time-based	1.36	0.15	1.59	0.28	0.49	Cutter #1
SFIIS-II	1.77	0.12	2.01	0.57	0.58	
LFBIIS	0.95	0.17	1.15	0.10	0.28	
Proposed	0.96	0.10	1.07	0.04	0.17	
Time-based	1.59	0.21	2.01	0.25	0.93	Cutter #4
SFIIS-II	1.24	0.17	1.49	0.27	0.69	
LFBIIS	1.28	0.07	1.38	0.29	0.48	
Proposed	1.17	0.07	1.26	0.19	0.38	
Time-based	1.10	0.201	1.38	0.14	0.47	Cutter #6
SFIIS-II	1.43	0.101	1.59	0.45	0.43	
LFBIIS	1.17	0.065	1.25	0.28	0.24	
Proposed	1.06	0.058	1.13	0.12	0.18	

**Table 6:** The benefits and challenges of the proposed method.

Benefits	1. Reduce or even eliminate the data loss
	2. Reduce or even eliminate the data redundancy
	3. Improve the problem of data imbalance
	4. Reduce the amount of sampled data
Challenges	1. Uncertainty risks caused by forecasting
	2. Accurate degradation expression, especially for feature indicators

## Data Availability

The dataset used in this paper is publicly available.
